# Effects of chemical warfare agent decontaminants on trace survival: Impact on fingermarks deposited on glass

**DOI:** 10.1111/1556-4029.15115

**Published:** 2022-08-23

**Authors:** Isabelle Radgen‐Morvant, Natalie Kummer, Christophe Curty, Olivier Delémont

**Affiliations:** ^1^ École des sciences criminelles University of Lausanne Lausanne Switzerland; ^2^ Federal Office for Civil Protection (FOCP) Laboratoire Spiez Spiez Switzerland

**Keywords:** CBRN, chemical weapons, cyanoacrylate, decontaminants, fingermarks, non‐porous surfaces, small particle reagent

## Abstract

Following a chemical incident involving chemical warfare agents or more broadly, chemical weapons, there are two possible approaches in dealing with the traditional forensic analysis of contaminated exhibits. The first is to analyze the contaminated items under safe conditions (i.e. in laboratories dedicated to the handling of such substances), while the second relies on item decontamination prior to processing them in traditional forensic laboratories. One of the main limitations of the latter is the possible degradation or destruction of traces caused by the decontamination process. Hence, it is crucial to have as much information as possible on the impact of different decontamination agents and procedures on traces. This research presents experimental results on the recovery of fingermarks on glass after the application of decontaminants typically used in case of chemical incidents. The impact of 11 decontaminants on fingermarks deposited on glass and on the subsequent enhancement with cyanoacrylate and Small Particle Reagent (SPR) was evaluated (by visual examination) by four evaluators. The results of the study demonstrated that the persistence of fingermarks on glass is highly dependent on the type of decontaminant used. Decontamination agents based on the principle of nucleophilic substitution to neutralize toxic chemicals allowed good subsequent development of fingermarks with SPR. Powdered decontaminants did not show any indication of alteration of fingermarks, whereas decontamination with oxidants leads to variable results.

Highlights

- The field of CBRN forensic is expanding, but a comprehensive approach for the handling of contaminated items is still lacking.
- This work highlights that post‐decontamination fingermarks may be recovered with traditional fingermark development technique as cyanoacrylate and small particle reagent.
- Understanding the impact of decontaminants on traces, and in this case, on fingermarks on glass, may help choose the best forensic development techniques to apply for contaminated (& decontaminated) items and develop procedures for the investigation of chemical events.

## 1 INTRODUCTION

Toxic chemicals may be released in the context of wars (e.g. the Syrian civil war [1]), terrorist attacks or criminal acts, such as the sarin gas attack on the Tokyo underground on 20 March 1995 [2], or the assassination of Kim Jong Nam with VX at Kuala Lumpur airport on 13 February 2017 [3]. The recent Novichok incidents in Salisbury in March 2018 and the attempted poisoning of Russian opponent Navalny in 2020 have shown that chemical weapons unfortunately continue to be in the headlines [4, 5]. In the various situations that involve the use of toxic chemicals to cause intentional harm, clarification of the modus operandi can provide crucial information, valuable both for the management and reconstruction of these events.

The contribution of forensic examinations is one of the fundamental aspects of understanding the modus operandi. In particular, examinations aiming at inferring the identity of protagonists can be important, such as the analysis of fingermarks or DNA. However, due to the toxicity of the chemical agent, the handling of objects collected from a contaminated scene is challenging. Two main strategies have been proposed so far. The first is to deal with the objects collected under safe conditions (i.e. in the glove boxes of laboratories dedicated to handling these substances). The second approach is to decontaminate the sampled items so that they can be analyzed in traditional forensic laboratories. The main disadvantage of this second approach is that decontamination procedures can lead to the alteration or even loss of the traces and the information they convey. It is therefore important to understand the impact of different decontamination procedures on traces of interest, such as fingermarks, to mention only the one included in this article.

The main methods of decontamination are based on the physical removal or chemical modification of chemical agents [6]. While physical methods aim to remove the toxic chemical by using, for example, water (possibly with soap) or an organic solvent (e.g. isopropanol), chemical methods modify the structure of the chemical to reduce or neutralize its toxicity.

Oxidation and nucleophilic substitution are the two main chemical reactions that can be used to neutralize a chemical warfare agent [6]. Oxidation is used to convert organophosphorus compounds (e.g. sarin) into the corresponding phosphonic acid, and sulfur‐based compounds (e.g. mustard gas) into the corresponding sulphoxide, sulphone and/or sulphonic acid. The oxidants used can be chlorine‐based substances, such as sodium hypochlorite (found in AllDecont), or less aggressive compounds that release active chlorine, such as chloramine (found in GD Universal) or sodium dichloroisocyanurate (found in BX24). Nucleophilic substitution aims to replace an atom (or group of atoms) to create a less toxic compound. Alkaline hydrolysis uses strong bases such as sodium hydroxide (NaOH) or potassium hydroxide (KOH), which are the active components in GDS2000 and GD‐6, respectively. In this case, the hydroxide ions HO^−^ disrupt and break the P–X bond(s) of the organophosphate nerve agents and form a P–OH bond instead. For mustard gas, the chlorine atoms are replaced by OH groups. The same is true for sarin and soman, where the fluorine atom is replaced by OH. Nucleophilic substitution of the chemical agent is also possible using oximes, such as 2,3‐butadione monoxime (the active compound of RSDL), which also results in the substitution of the fluorine atom (e.g. in sarin) by a hydroxyl group (OH) [6].

Decontamination by powdered absorbents, such as FastAct and CH‐Powder, is another approach that combines physical removal (via absorption capabilities) and chemical action (via hydrolysis and/or oxidation) of the decontamination sorbent [6]. The absorbent powder used can be removed within seconds of application, which can limit the contact time between the decontaminant and the fingermarks.

The impact of decontamination procedures on fingermarks depends on the mechanism (physical or chemical by oxidation or nucleophilic substitution) and the active compounds involved. It also depends on the composition of the existing fingermark. Although complex and variable, the natural composition of fingermarks includes amino acids, proteins and substances from sebum, such as fatty acids, glycerides, wax esters, squalene, sterols and sterol esters [7, 8, 9, 10]. Since fingermarks are often latent, various development techniques can be used to enhance the contrast between the trace and its substrate. Cyanoacrylate fuming (CA) and small particle reagent (SPR) are two of them, suitable mainly for enhancement on non‐porous surfaces. CA is the method of choice for enhancing fingermarks on glass. However, its application on fingermarks that have been exposed to water can be problematic, as parts of the hydrophilic components of the marks, acting as initiators of CA polymerization, can be washed away [7, 10]. Due to its reaction with insoluble components of fingermarks, SPR is described as the technique of choice for improving fingermarks on non‐porous surfaces that have been in contact with aqueous solutions [7, 11].

Physical removal with a polar solution (e.g. water, soap water or isopropanol) removes, in theory, mainly hydrophilic components of the fingermarks, such as amino acids, peptides and proteins. The hydrophobic components of the sebum (e.g. fatty acids and glycerides), if preserved, could serve to enable SPR development. Regarding chemical decontamination approaches, in brief, it seems that oxidants that release hydroxide ions (OH^−^) [12] and hypochlorite ions (ClO^−^) [9, 13] could react with the double bonds of unsaturated lipids (such as fatty acids, triglycerides, wax esters and squalene), as well as with the side chain and amino acid backbone of proteins [14]. In addition, strong bases (i.e. NaOH and KOH) used for alkaline hydrolysis of chemical agents could induce esterification of lipids with ester functional groups (e.g. glycerides). Hydrolysis of proteins would be less problematic as it leads to amino acids, but proteins could still lose their three‐dimensional structures (denaturation) and become insoluble under these conditions [15].

To our knowledge, only Wilkinson et al and Zuidberg et al have published studies comparing the impact of chemical agents and decontamination procedures on fingermarks [16, 17, 18]. Wilkinson et al first studied the impact of chemical agents on fingermarks, and then on some enhancement techniques, notably 1,8‐Diazafluoren‐9‐one (DFO), and Ninhydrin for porous surfaces, besides cyanoacrylate fumigation and powders for non‐porous surfaces. The effect of sarin and decontamination with the chlorine‐based decontaminant (CASCAD) on the enhancement of fingermarks was briefly investigated in an additional experiment [16]. More recently, in an international, inter‐laboratory study conducted by Wilkinson et al, sebum‐rich and bloody fingermarks deposited on paper and Ziplock plastic bags were exposed to various decontaminants (i.e. VHP, gamma irradiation, ozone, dry fogging, formaldehyde, chlorine dioxide, MODEC MDF‐500 and Bioxy‐S). After decontamination, several usual fingermark enhancement techniques, including amino acid reagents, blood reagents and cyanoacrylate, were applied. VHP and gamma irradiation had the least detrimental impact on the recovery of fingermarks, considering all reagents tested [17]. Zuidberg et al analyzed the effects of five decontamination procedures (which included physical removal with soap water and chemical decontaminants) on fingermarks before and after enhancement with vacuum metal deposition (VMD) techniques. They found that fingermarks could still be successfully enhanced using VMD in at least 70% of cases [18]. The results of these studies show that, although all the tested decontaminants decreased the quality of the fingermarks in general, in all cases some of them were still of sufficient quality to allow comparison for identification purposes.

Based on these considerations, the fundamental objective of our research is to expand the knowledge on the impact of decontaminants and decontamination procedures on the survival of fingermarks and to assess their effect on development techniques. To this end, experiments complementary to those of Zuidberg et al were conducted. Additional decontaminants and other types of decontaminants (e.g. powdered absorbents) were included, and we considered other enhancement techniques (i.e. cyanoacrylate fumigation and small particle reagent). In brief, all fingermarks were observed, photographed and assessed at three different stages: before decontamination, after decontamination and after the use of the development techniques (CA and SPR). The impact of the decontamination procedure itself was assessed by comparing the quality of the whole fingermarks before and after decontamination, while the ability of CA and SPR to enhance fingermarks after decontamination was considered by comparing the fingermarks after decontamination with the corresponding half‐fingermark after enhancement using respectively CA and SPR.

## 2 MATERIALS AND METHODS

**FIGURE 1:**
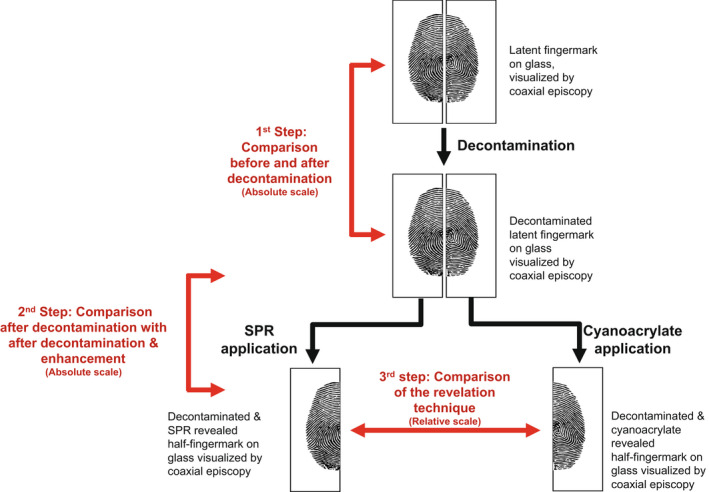
Schematic overview of the experimental design

The first objective of this study was to observe and evaluate the impact of 11 decontaminants on natural fingermarks deposited on glass. This was based on the comparison of the quality of fingermarks before and after decontamination (Figure 1, 1st step). The second objective was to assess the ability of two common development techniques (CA and SPR) to enhance the visualization of fingermarks present on glass surfaces after decontamination procedures. To do this, two distinct comparisons were made. First, the fingermarks (entire) before the development were compared with their corresponding fingermarks after CA (the half‐fingermark) or SPR (the counter‐half) enhancement (Figure 1, 2nd step). Second, the fingermark halves enhanced by CA were directly compared with their corresponding counter‐halves enhanced by SPR (Figure 1, 3rd step). These comparisons were used to determine which development technique was most suitable according to the decontaminant or the type of decontaminant applied. Entire fingermarks (deposed on two adjoining glass slides) were observed and photographed with a Leica Z6 APO microscope coupled to a Canon EOS 600D camera and using episcopic coaxial illumination (Light source Leica CLS 150X). Fingermarks were photographed before and after decontamination, as well as after CA and SPR development.

### 2.1 Fingermark deposition

A group of ten volunteers (seven women and three men aged between 25 and 41 years) was at the origin of the latent fingermarks used. For each of the 11 decontaminants considered, six or seven of the volunteers were asked to deposit three fingermarks (index finger, middle finger and ring finger) in the middle of two adjoining glass slides (Corning® microscope slides 2947 75 × 25 mm). This resulted in 18–21 fingermarks per decontaminant. Donors were asked to refrain from washing hands 30 minutes prior to the deposition and to gently rub the middle three fingertips of each hand together to ‘homogenize’ the secretion residues. The fingermarks were stored in microscope slide boxes for 2 days before being subjected to decontamination.

### 2.2 Decontamination

Eleven decontaminants commonly used by first responders or that were commercially available were selected and tested (Table 1). Three are based on the physical removal of the chemical agents (i.e. water, soap water and isopropanol), while six of them (Alldecont, RSDL, GDS 2000, GD‐6, GD Universal and BX24) aim to neutralize the chemical agent through chemical reactions. The last two ones (FastAct and CH‐Powder) use powder particles to absorb the chemical agent in liquid form (physical removal) and further neutralize the chemical agent (even when the powder is removed from the surface).

**TABLE 1:** List of the decontaminants tested with the indication of the manufacturer, the mechanism of action (and the type of compounds involved), and the list of components (with the active substance indicated in italic & Bold)

Mechanism Type of compounds	Decontaminant (manufacturer) Components with the active(s) substance(s)	Exposition time
Physical removal Polar solution	Isopropanol (Sigma‐Aldrich >99.8%) Isopropanol	Continuous flow for 2 min
	Soap Water (tap water with ‘Handy’ hand‐washing detergent) Water, ***anionic surfactants, amphoteric surfactants***, non‐ionic surfactants, fragrances, preservatives	Continuous flow for 2 min
	Water (from the tap) Water	Continuous flow for 2 min
Oxidation Chlorine‐based compounds	Alldecont (OWR) ***Sodium hypochlorite (NaOCl)***, stearic acid, capri acid, butyldiglycol	1 min
	BX24 (Cristanini) ***Sodium dichloroisocyanurate (NaDCC)***	15 min
	GD Universal (OWR) 2‐(2‐Butoxyethoxy) ethanol, ***Chloramine‐T***	2 × 10 min
Nucleophilic substitution Alkaline hydrolysis Strong base	GD‐6 (OWR) ***Potassium hydroxide (KOH)***, aminoethanol, benzylalcohol, propanol	15 min
	GDS 2000 (Kärcher) ***Sodium hydroxide (NaOH)***, 2‐Aminoethanol, diethylenetriamine, 2‐Amino‐natriumbutanolat, 1‐Butanol	10 min
Nucleophilic substitution Oximes	RSDL (Emergent Bio) ***2,3 butandion monooxime***, polyethylene glycol monomethyl ether, water	2 min
Absorption Hydrolysis and oxidation Powder	CH‐Powder (LBA) Chlorinated lime (CaCl_2_), magnesium oxide (MgO), ***formation of chlorine***	90 s for absorption
	FastAct (Enware) ***Magnesium oxide (MgO), titanium dioxide (TiO***_***2***_ ***)***	90 s for absorption

The physical removal of agent using water and soap water was achieved using a submersible water pump for fountains that creates a constant flow. The physical removal with isopropanol was performed using a laboratory wash bottle. Glass slides were placed in a slightly inclined position in a metal basket that allows the liquid to drain off. Glass slides were exposed to the flow of liquid for 2 min. Finally, glass slides were allowed to dry overnight.

For all the other decontaminants, glass slides were deposited in glass trays with the fingermarks facing up. The powdered decontaminants (FastAct and CH‐Powder) were added until the glass slides were completely covered. The powder was removed after 90 seconds, according to the manufacturer's instruction. Powder residues, if present, were washed out using a nitrogen flow. Liquid decontaminants (i.e. AllDecont, GSD2000, GD‐6, GD Universal and BX24) were sprayed onto the glass slides until they were completely covered. For RSDL, the sponges that contain the decontamination gel were squeezed on the glass slides. Fingermarks were passively exposed to the decontamination solutions for a period of time (varying from one decontaminant to another) determined by the manufacturer's instructions (between 1 and 20 min) (Table 1). Finally, glass slides were allowed to dry overnight.

### 2.3 Development of fingermarks

After decontamination, fingermarks development was carried out by cyanoacrylate fuming (CA) and small particle reagent (SPR). Each fingermark deposited in the middle of two adjacent glass slides was divided. One half was developed by CA, while the counter‐half was developed by SPR. To avoid bias, the side (left or right) of the fingermark treated with SPR or CA was randomly assigned for each fingermark.

#### 2.3.1 Cyanoacrylate fuming

A MVC® 1000‐D2 fuming cabinet (Foster + Freeman Ltd.) was used with Cyanobloom (Foster + Freeman Ltd.) as the CA monomer. Cyanobloom (0.8 g) was placed on an aluminium cup in contact with the heater set at 120°C. The cabinet humidity was fixed at 80%. Fingermarks were exposed to the CA fuming until a sufficient development was observed by the operator (fuming time was 10 min on average and up to 15 min).

#### 2.3.2 Small particle reagent

Glass slides were immersed (with the fingermarks facing up) for 30 s in the SPR bath (freshly made solution of molybdenum sulphide, docusate sodium and water) [19]. Further immersion of 30 s was carried out if necessary, until sufficient development had taken place. Specimens were then washed with distilled water and air dried.

### 2.4 Quality assessment of fingermarks

The quality of each fingermark or half‐fingermark was separately assessed by four evaluators from the images recorded. The entire fingermarks were evaluated before and after decontamination using the absolute grading system published by the Center for Applied Science and Technology (CAST) [20, 21]. For each fingermark, a quality score ranges from 0 (no ridge detail) to 4 (complete ridge detail). These scores were used to evaluate the impact of the decontamination procedures on the fingermarks.

The quality of each fingermark before development (but after decontamination) and of its corresponding marks after development with CA (the half‐fingermark) or SPR (the counter‐half) was scored using the same system. These scores were used to study the benefits of development techniques (i.e. CA and SPR) applied to decontaminated fingermarks.

A relative grading system (ranging from −2 and 2) developed by the University of Canberra, Australia, was used to directly compare the development with CA and SPR [21]. All the fingermarks enhanced half with CA and half with SPR were scored by the 4 evaluators. When a significant increase in quality was observed for one of the half‐fingermarks, a score of 2 (quality after SPR development is significantly better than after CA development) or −2 was given (the quality after CA development is significantly better than after SPR development). The zero (0) value was assigned when no difference regarding the quality of the two half fingermarks was observed. In addition, a minor increase in the quality was graded 1/−1. Finally, the value 00 was given if on neither side (right and left) fingermarks were visible.

To avoid bias, the images of fingermarks before and after decontamination from all the donors appeared to the evaluators in a random order, and without any indication. For the comparison of the half fingermarks developed by CA or by SPR, the disposition (left or right) for SPR and CA half‐fingermark was random and variable.

### 2.5 Data treatment

As the quality of the fingermarks was graded by four evaluators, each fingermark received four score values. Instead of taking the average for the further calculations, it was decided to use these four values for the data treatment. This induces that each fingermark has four scores and that in the subsequent calculations these four scores are considered.

The percentage of fingermarks considered (using the absolute grading system) as suitable for identification purposes (i.e. score equal to 3 or 4), as much as the percentage of fingermarks that are visible but not necessarily suitable for identification (i.e. score 2) and the percentage of fingermarks that are not visible or almost not visible (i.e. score 0 or 1) were calculated.

#### 2.5.1 Quality of the fingermarks

The quality of fingermarks after deposition was estimated observing the scores (using the absolute grading system) of fingermarks before decontamination. Donors having more than 90% of their fingermarks suitable for identification (i.e. score equal to 3 or 4) were considered “good donors”. Following the same principle, donors having between 80% and 90% of their fingermarks suitable for identification purposes were considered “medium donors”. Finally, donors with less than 80% of their fingermarks suitable for identification purposes were classified as “bad donors”.

#### 2.5.2 Impact of decontamination procedures

The impact of decontaminants was estimated by comparing the scores of each fingermark before and after the decontamination (using the absolute grading system). The percentage of fingermarks considered as suitable for identification purposes (score of 3 or 4), as visible but not necessarily suitable for identification (score of 2) and as (almost) not visible (score of 0 or 1) were calculated for each of the 11 decontaminants (Figure 1, 1st step).

#### 2.5.3 CA and SPR developments of decontaminated fingermarks

The ability of cyanoacrylate fuming (CA) and small particle reagent (SPR) to enhance decontaminated fingermarks was evaluated in two steps. First, the score (using the absolute grading system) of each fingermark after decontamination was compared with the score obtained for the corresponding fingermarks after development with CA (the half‐fingermark) or SPR (the counter‐half). In the same way as for the previous evaluation of the impact of the decontamination, the percentage of fingermarks in each quality category was computed (Figure 1, 2nd step).

In a second step, each half‐fingermark enhanced with CA was compared to its counter‐half enhanced by SPR using a relative grading system. The score obtained using the relative grading system (ranging from −2 and 2) indicated which development technique could be more favorable to apply depending on the decontaminant used (Figure 1, 3rd step).

## 3 RESULTS AND DISCUSSION

**FIGURE 2:**
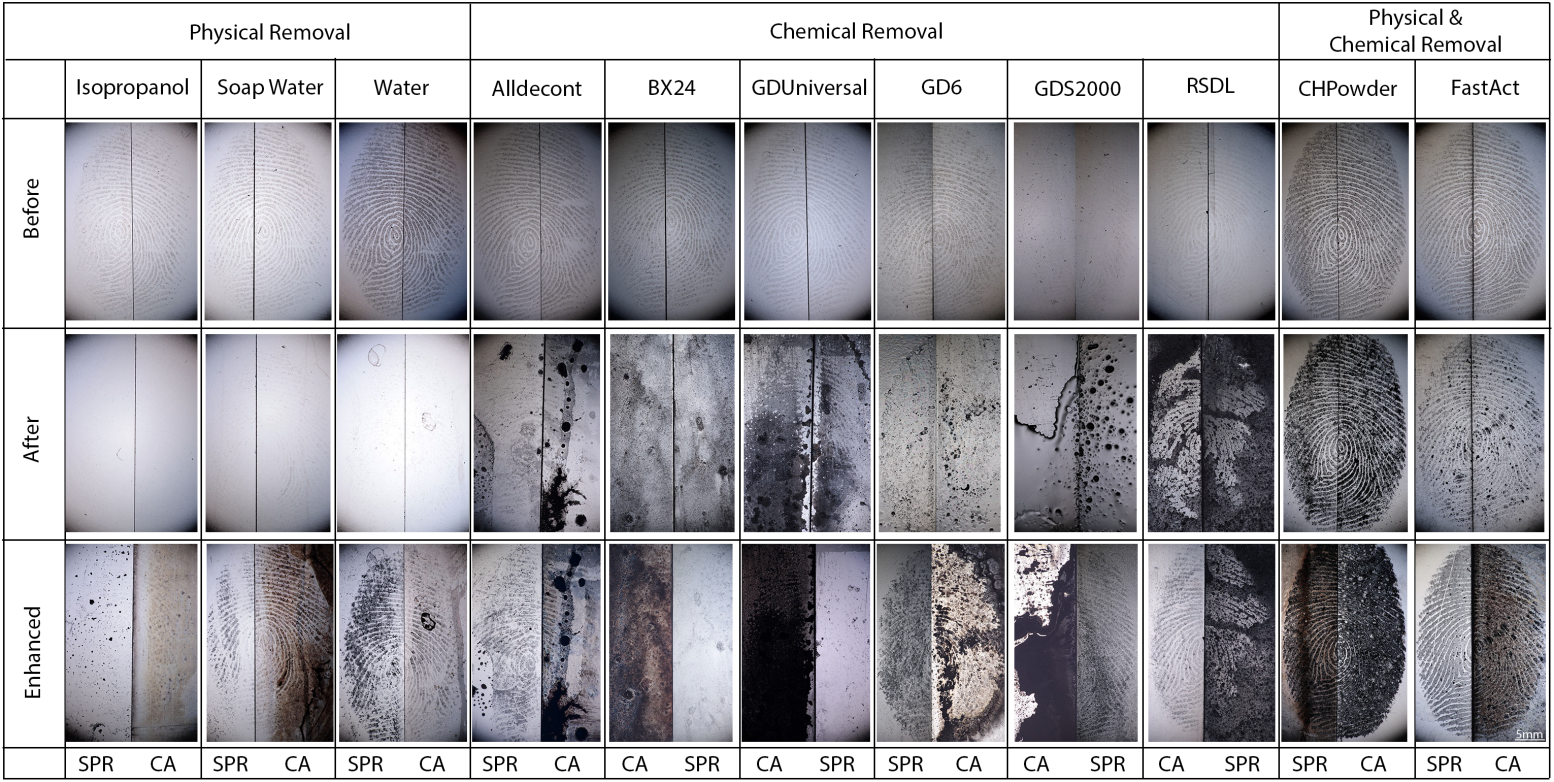
Overview of fingermarks from one donor index, for each decontaminant and at each step. From top to bottom: fingermarks before decontamination, after decontamination and enhanced fingermarks. The enhancement techniques applied on each half‐fingermark is mentioned under each corresponding half‐fingermark

For each of the 11 decontamination procedures tested, fingermarks from six to seven donors (three fingermarks per donor) were deposited in the middle of two adjoining glass slides. This was done by four evaluators examining the photographs taken before any treatment. Having more than 90% of their fingermarks suitable for identification, four donors were considered “good donors”. Following the same principle, two of them were qualified as “medium donors” (between 80% and 90% of their fingermarks suitable for identification purposes), while four of them were qualified as “bad donors” (less than 80% of their fingermarks suitable for identification purposes). Fingermarks were observed and photographed (a) after the deposition (before decontamination), (b) after decontamination (before development) and (c) after development with CA (the half‐fingermark) or SPR (the counter‐half). The quality of fingermarks was assessed at these three stages, providing an evaluation of the impact of decontamination procedures on fingermarks and the ability of CA and SPR to enhance fingermarks after decontamination (Figure 2).

### 3.1 Quality of fingermarks

The quality of the fingermarks provided by the ten donors was assessed by calculating the percentage of fingermarks suitable for identification purposes (i.e. score equals to 3 and 4 using the absolute scale). For each decontaminant, on average 75% of fingermarks involved were scored as suitable for identification purposes (results range between 60% and 87%) before decontamination. It is important to underline that the quality of fingermarks at this stage was solely based on the images taken through optical examination.

A natural deposition process, producing fingermarks of variable quality, was chosen as in a real case scenario; traces may be of poor quality and partial. To be able to compare results, fingermarks were observed and photographed under standardized lighting and photographic conditions.

### 3.2 Impact of decontamination procedures on fingermarks

**FIGURE 3:**
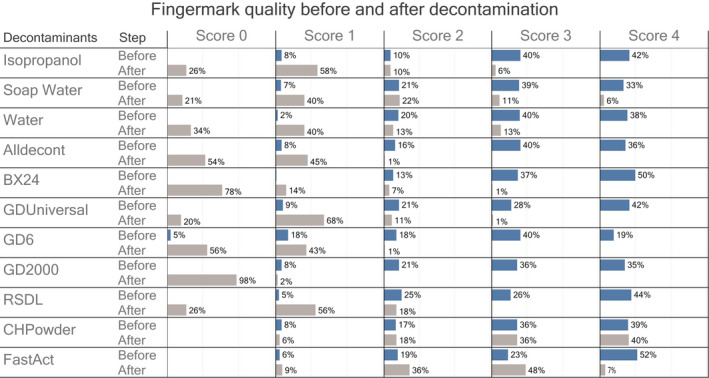
Effect of decontamination procedures on the quality of the ridge detail of latent fingermarks (Figure 1, 1st step). On the right, the names of the different decontaminant materials and the stage of evaluation (i.e before and after decontamination). The colored portions illustrate the distribution of the quality scores (0–4) within the different groups (i.e blue before decontamination and gray after decontamination). Value 0 corresponds to “No evidence of mark”, value 1 corresponds to “weak development; evidence of contact but no ridge details”, value 2 corresponds to “limited development: about 1/3 of ridge details are present but probably cannot be used for identification purposes”, value 3 corresponds to “strong development; between 1/3 and 2/3 of ridge details; identifiable fingermark”, and value 4 corresponds to “very strong development; full ridge details; identifiable fingermark”

The impact of the decontamination procedure on fingermarks was assessed by comparing the score (using the absolute scale) of each trace before and after decontamination without any enhancement using episcopic coaxial illumination. The impact was found to be highly dependent on the decontaminant used, as illustrated by the results in Figure 3.

The decontaminants under powder form (CH‐Powder and FastAct) had the least deleterious effects. The percentage of fingermarks with high quality (i.e score 3 & 4) after decontamination was 76% for the CH‐Powder and 55% for the FastAct (before the decontamination these values were 75% for both). Overall, the effect was almost non‐existent for CH‐Powder and rather weak for the FastAct. For the traces treated by the latter, a general alteration of the quality of the deposits was observed, which impacted the potential of exploitation of some of these traces in a comparative identification approach but did not increase the rate of (almost) not visible traces (i.e. score of 1 or 0).

For all the other commercial decontaminants, which were all liquids or gels, the degradation of the quality of the fingermarks was substantial, with 82%–100% of the fingermarks being not or almost not visible (i.e. score of 1 or 0) after decontamination. AllDecont, GD‐6 and GDS2000 have practically wiped out all traces, with less than 1% of fingermarks remaining visible (i.e. score 2–4) after decontamination. BX24, GD Universal and RSDL were a little less damaging for the fingermarks, with, respectively, 8%, 12% and 18% of them still visible and eventually exploitable after the decontamination (i.e. score 2–4).

These observations can be compared with the results obtained by Zuidberg and colleagues [18] who reported a ratio of about ~30% (estimated from Figure 2 in [18]) of fingermarks being visible (i.e. score equals to 2, 3 or 4) after decontamination by GDS2000. They also reported that SDF—a decontaminant with the same active principle (NaDCC) as BX24—resulted in about 10% of fingermarks still visible. We observed that decontamination with BX24 led to 8% of fingermarks still visible or exploitable (i.e. score 2–4). Similar trends were found for the bleach‐based solution tested by Zuidberg and colleagues, and the decontaminant containing NaOCl that was considered in our tests (i.e. AllDecont). In both studies, a very large proportion of the fingermarks were altered by these decontaminants.

Physical removal with water, soap water or isopropanol also had a negative impact on the quality of the fingermarks, resulting in a significant proportion of fingermarks being (almost) not visible. The percentage of fingermarks still visible and exploitable after the decontamination (i.e. score 2–4) were 26% for water, 39% for soap water and 16% for isopropanol. These rates are much lower than the ones published by Zuidberg and colleagues, who reported around 70%–90% of fingermarks are still visible and exploitable after the decontamination by (soap‐)water. This difference can be accounted for by the application conditions. While Zuidberg and colleagues maintained the glass slides in a bath, we exposed the specimens to a continuous flow of liquid, as to wash away contaminants.

In a second look, these results provide the first knowledge to better understand and predict the impact of different decontaminants type on fingermarks. The two decontaminants that contain strong bases (sodium hydroxide for GD‐6 and potassium hydroxide for GDS2000), and the decontaminant that contain sodium hypochlorite as oxidants (i.e. AllDecont) were the ones that had the greatest negative impact on the quality of fingermarks. By contrast, decontaminants that contain less aggressive oxidants (i.e. sodium dichloroisocyanurate for BX24 and chloramine for GD Universal) were found to be somewhat less damageable for fingermarks on glass.

### 3.3 Ability of CA and SPR to enhance decontaminated fingermarks

**FIGURE 4:**
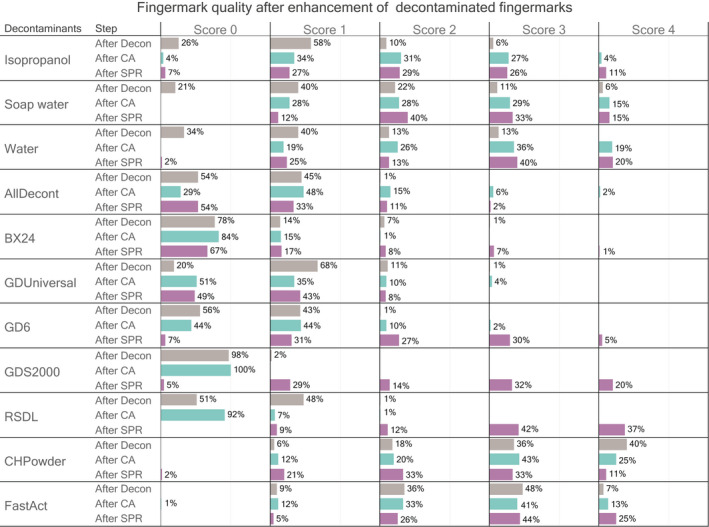
Quality (ridge details) of decontaminated fingermarks directly after decontamination (in gray), and after application of development technique (CA and SPR, respectively, in light blue and purple). On the right, the names of the different decontaminant materials and the stage of evaluation. The colored portions illustrate the distribution of the quality scores (0–4) within the different groups. Regarding the scores, 0 means no ridge detail observed using episcopic coaxial illumination and 4 all ridge detail visible

**FIGURE 5:**
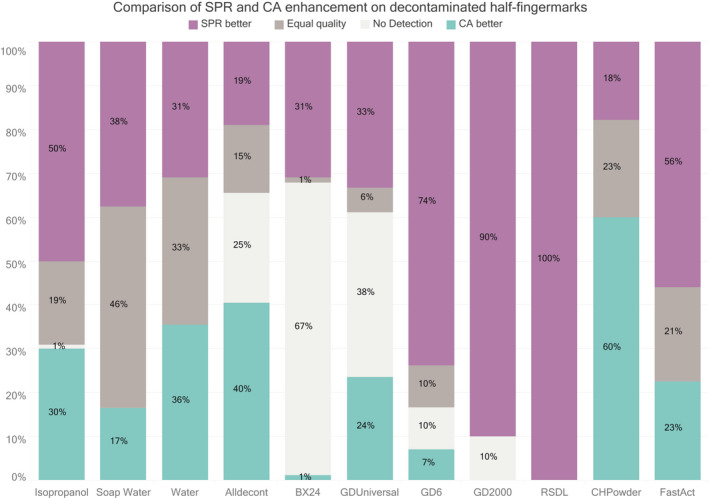
Distribution of the relative scores obtained for the fingermarks after decontamination, and after the development with CA (half‐fingermark) or SPR (counter half‐fingermark). Relative score of −1 or −2 means that the quality of the half fingermark enhanced by CA was considered as better than the quality of the counter half enhanced by SPR (blue color). Relative score of 1 or 2 means that the quality of the half fingermark enhanced by SPR was considered as better than the quality of the counter half enhanced by CA (purple color). A relative score equals to 0 means that no quality difference between the half‐fingermarks enhanced by CA or SPR was detected (gray color). Fingermarks that are not visible on neither side are also indicated (white color)

The ability of two common development techniques, cyanoacrylate fuming (CA) and small particle reagent (SPR), to enhance fingermarks previously submitted to decontamination was evaluated using two approaches. First the score assigned to each fingermark after decontamination was compared with the score obtained for the corresponding half‐fingermarks after development with CA or SPR (Figure 4). Second, each half‐fingermark enhanced by CA was compared to its counter‐half enhanced by SPR using a relative grading system (Figure 5).

For fingermarks that have been decontaminated using isopropanol, soap water or water, the development with CA or SPR increased the overall quality of the half‐fingermarks. The ratio of fingermarks described as exploitable (i.e. score 2–4) after the decontamination and subsequent development technique reached 62% (isopropanol), 72% (soap water) and 81% (water) for CA, and 66% (isopropanol), 88% (soap water) and 73% (water) for SPR. These values are close to the percentages describing the quality of the fingermarks before decontamination procedures. It was observed that for fingermarks from good donors, the decontamination had even a positive effect, as it washed away the excess secretions giving the possibility to have a more defined fingermark and a better resolution of ridge details.

After the decontamination procedure with AllDecont, the application of development techniques produced a quality gain: the percentage of fingermarks scored as exploitable increased from 1% to, respectively, 23% (CA) and 13% (SPR).

The quality of fingermarks decontaminated with GD‐6, GDS2000 and RSDL was improved by the development with SPR, but not (or only slightly) by CA. After the decontamination the percentages of fingermarks scored as exploitable (score 2–4) were 0%–1% for these three decontaminants. After SPR treatment, this percentage increased to 62%, 66% and 91%, respectively. A similar trend, although of lesser magnitude, was observed for BX24, with an increase in exploitable fingermarks from 8% to 16% after development by SPR.

The quality of the traces could not be improved upon decontamination with GD Universal by CA or SPR development techniques.

CA development did not seem to impact (neither positively nor negatively) the quality of fingermarks that were previously decontaminated by CH‐Powder or FastAct. For fingermarks decontaminated with CH‐Powder, a decrease in the percentage of fingermarks suitable for identification (score 2–4) was noted after SPR development (from 94% to 77%), while this development technique led to a slight increase for fingermarks decontaminated with FastAct (from 91% to 95%). To date, we found no reliable explanation for this opposing trend. We hypothesize that powder residues remaining in the valley of the fingermarks could affect the quality by reacting with SPR. However, we were not able to test this hypothesis so far.

In general, these results suggest that SPR development should be favored after decontamination procedures relying on nucleophilic substitution (alkaline hydrolysis and oximes). The target compounds of SPR (which are the hydrophobic fractions of fingermarks) have been apparently preserved. At least in a way that has allowed SPR enhancement of some fingermarks, although we might have expected that the alkaline conditions created by GDS2000 and GD‐6 could have a detrimental impact on lipids. Two main hypotheses appear here, the target compounds (or a part of them) were not hydrolysed and/or the products of the hydrolysis (the corresponding sodium or potassium salt) are still targets of SPR.

The limited success of fingermarks enhancement using CA is, however, probably not induced by the disruption of target compounds but rather due to an excess of them. CA polymerization is supposed to occur through an anionic reaction with anionic initiator sites created through (OH^−^) ions [7, 10]. It was also shown by Wagacki et al. (2007) that a more basic environment favors CA polymerization. In this case, the excess of initiator sites and the favorable conditions for polymerization may lead to an overdevelopment of CA polymers and lead to complete coating of the decontaminated items.

The use of an alkaline solution may therefore be problematic with CA enhancement as the residues of the decontaminants probably create numerous initiator sites for the CA polymerization and lead to a complete coating of CA on the glass slides (Figure 2). It can be observed that for some decontaminants (e.g. BX24), due to the overall deposition of CA on the glass slide which creates widespread background noise, no successful observation of ridge details through coaxial episcopic lighting was possible. An additional rising step after decontamination could help reduce background noise but also increase loss of target compounds for fingermarks enhancement.

Results of this study have shown that decontaminants that used oxidizing agents, mainly chlorine based ones, as active principle have a high impact on fingermarks. Chemically speaking, hypochlorite (ClO^−^) ions present in a solution may induce the oxidation of amino acids, peptides, and proteins as well as of unsaturated lipids [9, 14]. The low percentage of fingermarks scored as exploitable (score 2–4) after the decontamination based on oxidative processes (1% for AllDecont, 8% for BX24 and 12% for GD Universal) are findings that support this hypothesis. In addition, a low percentage of decontaminated fingermarks was scored as exploitable after the enhancement with SPR or CA for this class of decontaminants. The oxidizing reactions may modify the composition of fingermarks, and chemically alter the target components for CA or SPR, partially or fully inhibiting the enhancement process. This seems particularly true for SPR development, after decontamination with solutions containing active chlorine, where the poor results in development speak for the alteration of the hydrophobic fraction of the fingermarks.

Regarding the CA development, the presence of nucleophilic initiator in the solution may create a multitude of CA polymerization initiator sites which may seriously disrupt the enhancement process, as already depicted. The background noise is more or less intense depending on the decontaminants, indicating that some mixture may create less disruptive elements for the enhancement. Although small, a slight increase in the percentage of fingermarks considered exploitable was observed after CA (after decontamination with AllDecont) or SPR development (after decontamination with AllDecont and BX24).

A more detailed study was undertaken on the development technique to be favored (CA or SPR) depending on the decontaminant used. Relative scores obtained for the complementary half‐fingermarks analyzed by CA and SPR were computed (Figure 5). First of all, these results confirmed the observation that after decontamination with GD‐6, GDS2000 or RSDL, SPR is clearly more efficient than CA to enhance fingermarks. After decontamination with CH‐Powder or AllDecont, the quality of fingermarks enhanced by CA tended to be better (in 60% of the cases for CH‐Powder and in 40% of the cases for AllDecont) than the one enhanced by SPR (better in 18% of the cases for CH‐Powder and in 19% of the cases for AllDecont). In contrast, a higher percentage of the fingermarks were better enhanced by SPR than by CA after a decontamination using FastAct (56% again 23%), isopropanol (50%–30%), soap water (38%–17%) or BX24 (31%–1%). For GD Universal and Water, no clear evidence of quality improvement by one development technique over the other was observed.

## 4 CONCLUSION

The impact of different decontamination procedures on the survival of fingermarks was tested using experiments with fingermarks deposited on glass slides. Fingermarks were observed and photographed two days after deposition (before decontamination), after decontamination and after development with Cyanoacrylate fuming (CA) or small particle reagent (SPR). The quality of fingermarks was assessed at these three stages by four independent evaluators.

It was observed that after decontamination, the persistence of fingermarks on glass is highly dependent on the type of decontaminant used. Decontamination agents based on the principle of nucleophilic substitution (i.e. GD‐6, GD2000, RSDL) allow good development of fingermarks with SPR. Powdered decontaminants did not show any indication of alteration of fingermarks, and in some cases, decontamination even contributed to increase the visibility of the traces. Decontamination with oxidants (i.e. Alldecont, BX24, GD Universal) lead to variable results with a strong decrease of the capacity of fingermark interpretation. Further studies would be necessary to understand the observed tendencies. Physical removal (with isopropanol, soap water, water) affected the fingermarks but further development with SPR or CA often resulted in good quality traces.

This study should be further extended to include porous substrates to get a more complete picture of the influence of decontamination procedures on the detection of fingermarks. This should bring a valuable contribution to the general understanding of forensic possibilities post decontamination. Similar studies should also be undertaken on other relevant forensic traces, obviously DNA, but also digital traces which are becoming increasingly important in investigations. Finally, the influence of applying a toxic chemical as a contaminant directly to the trace, prior to decontamination, should be included in a next study.

## CONFLICT OF INTEREST

All authors declare that they have no conflicts of interest.
